# Towards Identifying Autistic Adults at Risk for Eating Disorders: A Brief Report Into Clustering of Social Camouflaging and Sensory Processing Differences

**DOI:** 10.1002/erv.70062

**Published:** 2025-12-02

**Authors:** Emy Nimbley, Siofra Bradley, Abigail Pickard, Michelle Sader, Ellen Maloney, Fhionna Moore, Tasha Suratwala, Helen Sharpe, Fiona Duffy, Karri Gillespie‐Smith

**Affiliations:** ^1^ School of Health in Social Science University of Edinburgh Edinburgh Scotland; ^2^ NHS Tayside Dundee Scotland; ^3^ School of Medicine Medicinal Sciences and Nutrition University of Aberdeen Aberdeen Scotland; ^4^ Leicestershire Partnership NHS Trust Leicester UK; ^5^ NHS Lothian Child and Adolescent Mental Health Services Edinburgh Scotland

**Keywords:** autism, eating disorders, sensory, social camouflaging

## Abstract

**Background:**

Autistic people with an eating disorder (ED) are at higher risk of poorer treatment outcomes and experiences, perhaps due to a lack of understanding surrounding underlying mechanisms. Several factors have been implicated, such as sensory processing and social camouflaging; however, there has been little empirical investigation into how such mechanisms group or cluster together, and if certain clusters place the individual at greater risk of ED severity.

**Method:**

A secondary data analysis was conducted on an online survey of *n* = 180 Autistic adults (mean age = 38 years). Participants completed self‐reported measures of sensory processing, social camouflaging and ED symptoms. Hierarchal clustering analyses (HCA) was conducted to explore clustering on sensory and social camouflaging behaviours, and a one‐way ANOVA was conducted to explore between‐cluster differences on ED symptoms.

**Results:**

Three distinct clusters were identified: Cluster 1 (high camouflaging, low sensory); Cluster 2 (high camouflaging, high sensory); and Cluster 3 (low camouflaging, average sensory). Participants in Cluster 2 reported significantly higher ED symptoms that those in Cluster 3. There were no significant differences between remaining clusters.

**Conclusion:**

Findings suggest the combination of these factors may place Autistic individuals at higher ED risk, although future longitudinal, mixed‐method and more representative research, which considers a wider range of risk mechanisms, is urgently needed before conclusions can be drawn.

## Highlights

- Using a data‐driven clustering approach, three clusters were identified in a large sample of Autistic adults: one with high camouflaging and low sensory, on with high camouflaging and high sensory, and one with low camouflaging and average sensory.
- Autistic adults in the high camouflaging and high sensory cluster had significantly higher eating disorder (ED) symptoms than those with high camouflaging and average sensory only.
- Despite limitations of cross‐sectional methods, results suggest a combination of social camouflaging and sensory difference may place an Autistic individual at greater ED risk, although future longitudinal, mixed‐method and more representative is warranted.

## 1 Introduction and Aims

It is estimated that between 25% and 28% of Autistic individuals report experiencing an eating disorder (ED) (Brown et al. 2024; Nuyttens et al. 2024; Westwood and Tchanturia 2017). Longitudinal studies have reported that Autistic traits in childhood predict disordered eating in adolescence (F. Solmi et al. 2021; Carter Leno et al. 2022), echoing experiences of Autistic women who discuss childhood Autistic traits interacting with a range of factors leading to the development of their ED (Brede et al. 2020). The extent to which this co‐occurrence represents a true overlap has been challenged through several lines of enquiry (Adams et al. 2024), particularly with regard to whether elevated rates are indicative of ‘true’ autism, or whether they are a secondary consequence of the ED. Evidence suggests elevated rates of Autistic traits reflect a combination of trait (reflective of ‘true’ autism) and state differences (Nuyttens et al. 2024; Kerr‐Gaffney et al. 2021; Sader et al. 2025). Importantly, those with higher Autistic traits and diagnosed Autistic individuals report similar clinical characteristics (Brede et al. 2024) and poorer ED treatment outcomes and experiences (Nimbley, Sharpe, et al. 2025).

One possible explanation for poorer ED treatment outcomes and experiences is a lack of understanding of the underlying mechanisms of EDs in Autistic individuals. Several autism‐specific factors have been implicated, including sensory and social differences (Brede et al. 2020; Adams et al. 2024; Loomes et al. 2025). Sensory differences have been reported early in life (Robertson and Baron‐Cohen 2017), continuing throughout development (Ben‐Sasson et al. 2019) and have been consistently reported as an integral part of Autistic experiences of EDs (Brede et al. 2020; Loomes et al. 2025). Additionally, social camouflaging (here defined as a behavioural strategy used to hide Autistic traits or adopt neurotypical traits during social interactions; Hull et al. 2017), has been recently suggested to play an important role (Nimbley, Buchan, et al. 2025). Indeed, a recent study explored sensory processing, Autistic identity and social camouflaging and found that social camouflaging was the sole predictor of ED symptoms in Autistic adults (Bradley et al. 2024). Collectively these findings suggest that sensory and social factors are key mechanisms in the overlap between autism and EDs, tentatively suggesting a prominent role of social camouflaging. However, given the heterogeneity of both EDs the Autistic experience (Meneguzzo et al. 2025), it is unlikely that there is a single pathway towards an ED in this population. Furthermore, while research to date has advanced our knowledge on putative mechanisms, little has been done in the way of trying to explore patterns of these mechanisms. Such findings would provide a strong rationale for conducting larger‐scale longitudinal studies, which over time will have clear implications towards improving ED outcomes in Autistic individuals, for example, through improved ED diagnostic accuracy and early intervention and prevention.

Data‐driven approaches, such as clustering analyses, are a promising method to explore possible subgroups for Autistic individuals with an eating disorder. Previous autism studies have successfully used such approaches on sensory and social interaction and communication differences, and to link such these clusters to clinical outcomes (e.g., Elwin et al. 2017; Kaneko et al. 2023; Husmann et al. 2024). Recent cluster analyses in eating disorder samples have also identified potentially ‘high‐risk’ profiles characterised by elevated autistic traits (Li et al. 2023; Meneguzzo et al. 2025).

Thus, this brief report aims to conduct exploratory cluster analyses on secondary data from Bradley and colleagues' (2024) study. Cluster analyses are data‐driven statistical approaches that can be used to begin exploring multi‐dimensional risk factors and to identify possible at‐risk subgroups within heterogenous populations (Gao et al. 2023). This approach has been suggested to be particularly relevant to the Autistic and ED community, where identifying subgroups may lead to improved, personalised autism‐affirming ED care (Meneguzzo et al. 2025). The study therefore aims to explore whether there are different clusters in sensory processing and social camouflaging behaviours in a sample of Autistic adults, and, if so, whether these clusters differ significantly according to their ED symptoms. Given the paucity of research in this area, there were no hypotheses about number of clusters, however it is hypothesised that cluster analysis will identify distinct subgroups of Autistic adults based on their social and sensory characteristics, and that these clusters will differ significantly in their levels of ED symptoms.

## 2 Methods

This is a brief report on a secondary analysis of data collected to explore the role of sensory processing, social camouflaging and Autistic identity in ED symptomology in a sample of Autistic adults (Bradley et al. 2024). The original study received ethical approval from the NHS West Midlands Solihull Research Ethics Committee and NHS Tayside. The amendment for secondary data analysis was approved by the University of Edinburgh School of Health in Social Science Research Ethics Committee (23‐24CLPS179).

### 2.1 Participants

To be eligible for the study, participants were required to be over the age of 18‐years‐old, fluent in English, and to have either a formal diagnosis or self‐identify as Autistic. The current sample recruited a mixture of both self and formally diagnosed Autistic individuals. This approach is in line with a more inclusive and neurodiverse‐affirming approach to research and clinical practice advocated for by the Autistic community (Fletcher‐Watson et al. 2019). This approach acknowledges significant barriers to assessment and reported waiting times of up to 8 years (Overton et al. 2023; Babb et al. 2021), reflecting a more inclusive approach to autism commonly adopted in clinical services to improve access to services (Brede et al. 2024).

Participants were recruited from the community through social media adverts, as well as internal university mailing lists and charity webpages. Participants were also recruited through the National Health Service (NHS), with clinicians in the NHS Tayside Eating Disorder service (TEDS) and Autism service (Tayside Adult Autism Consultancy Team; TAACT) informing eligible participants about the study. The recruited sample of 181 participants were mostly female (64.4%) and white (87.2%). 65 (36.1%) of participants reported a range of EDs (see Table 1 for full participant characteristics). The number of participants satisfied sample size calculations for the hierarchal clustering analyses, allowing for stable and reliable clustering with samples < 150 (Dalmaijer et al. 2022).

**TABLE 1:** Percentage and means and standard deviations of study characteristics and measures (*n* = 180).

Characteristics/measures		*N* (%)
Gender		
	Female	116 (64.4)
	Male	36 (20)
	Transgender	19 (10.6)
	Prefer not to say	9 (5)
Ethnicity		
	White	157 (87.2)
	Mixed or multiple	12 (6.7)
	Black or African American or Caribbean	5 (2.8)
	Asian	5 (2.8)
	Other	1 (0.6)
Autism diagnosis		
	Formal diagnosis	145 (80.6)
	Self‐identification	35 (19.4)
Eating disorder diagnosis		
	No	115 (63.9)
	Yes	65 (36.1)
Eating disorder diagnosis (*n* = 65)		
	Multiple	23 (35.3)
	Anorexia nervosa	22 (33.8)
	Bulimia nervosa	6 (9.2)
	Binge eating disorder	5 (7.7)
	Other	4 (6.2)
	Other specified feeding and intake disorder (OSFED)	4 (6.2)
	Avoidant and restrictive feeding intake disorder (ARFID)	2 (3.1)
	Not disclosed	1 (1.5)
Other mental health diagnosis/es		
	Yes	116 (64.4)
	No	64 (36)

		*M* (SD)
Age (*n* = 8 missing)		38 (13.6)
Eating disorder symptoms		6.86 (4.86)
Sensory processing (SPQ‐10)		19.74 (6.37)
Social camouflaging (CAT‐Q)		115.48 (17.99)

Abbreviations: CAT‐Q, Camouflaging Autistic Traits Questionnaire; EDEQ‐S, Eating Disorder Examination Questionnaire Short; SPQ‐10, Sensory Processing Quotient.

### 2.2 Procedure

Interested participants followed the link provided in the social media adverts or by clinicians to an online survey (Qualtrics; https://www.qualtrics.com/en‐gb/). They were then shown an information sheet and asked to provide written consent. Once consent was obtained, participants were asked to provide demographic information and complete a series of measures exploring sensory processing, social camouflaging, Autistic identity and ED symptoms. At the end of the survey, participants were shown a debrief sheet with signposting to further information and support resources. No financial compensation was offered for participation.

### 2.3 Measures

#### 2.3.1 Social Camouflaging

The Camouflaging Autistic Traits Questionnaire (CAT‐Q; Hull et al. 2019) was used to measure levels of social camouflaging behaviours. It is a self‐report questionnaire that has 25 statements, with participants responding on a 7‐point Likert scale ranging from Strongly Agree to Strongly Disagree. The maximum total score is 175, with higher scores indicating more camouflaging behaviours. The CAT‐Q has three subscales exploring specific strategies: compensation ‐ strategies used to actively compensate for difficulties in social situations (9 items), masking ‐ strategies used to hide Autistic characteristics (8 items), and assimilation ‐ strategies adopted to try and fit in with others during social interactions (8 items). The total CAT‐Q showed robust internal consistency in the current study (*α* = 0.82; *ω* = 0.92). Good reliability was also found across compensation (*α* = 0.88; *ω* = 0.88), masking (*α* = 0.85; *ω* = 0.87) and assimilation (*α* = 0.80; *ω* = 0.79) subscales.

#### 2.3.2 Sensory Processing

Sensory differences were measured using the 10‐item Sensory Processing Quotient (SPQ‐10; Greenberg et al. 2018). The SPQ‐10 asks participants about sensory differences across the five sensory modalities (smell, taste, sight, touch, sound), asking them to rate their responses on a four‐point scale between strongly agree, slightly agree, slightly disagree and strongly disagree. There is a maximum score of 30, with higher scores indicating higher sensory sensitivities. Good internal consistency (*α* = 0.85; *ω* = 0.86) was shown in the current study.

#### 2.3.3 Eating Disorder Symptoms

The Eating Disorder Examination Questionnaire Short (EDE‐QS; Gideon et al. 2016) was used to measure levels of ED symptoms. The measure asks participants to indicate how often they have exhibited a certain ED behaviour or cognition in the past 7 days on 12 items. Responses are scored along a four‐point scale of 0 days, 1–2 days, 3–5 days and 6–7 days respectively. Higher scores indicate more severe ED symptomology, with a maximum score of 36 and a clinical cut‐off of 15 and above (Prnjak et al. 2020). The EDE‐QS displayed high internal consistency in the current study (*α* = 0.91; *ω* = 0.92).

### 2.4 Cluster Analyses

Data was inspected for outliers (values > +3 or < −3 of standardised z‐scores). Marginal outliers were detected, and clustering analyses were run with and without outliers to compare dendrograms (visual representations of clustering) and cluster solutions. Outliers were determined not to make an impact on clusters and thus were not removed from analysis.

An exploratory hierarchal cluster analysis (HCA) using Ward's clustering method was conducted on total CAT‐Q and SPQ‐10 scores to explore the number of clusters. HCA is a bottom‐up approach which repeatedly combines similar responses based on closeness (here set as squared Euclidean Distance as all variables were continuous) until all data merged into a singular, final cluster. Dendrograms were inspected to determine three clusters. A one‐way ANOVA was run to compare clusters on age and chi‐square tests were run to compare clusters on gender. A one‐way ANOVA was then conducted to explore between‐cluster differences on eating disorder symptoms, as measured by EDE‐QS scores, with Tukey's post hoc tests run to test for the direction of significant differences (*p* = 0.05). Data were analysed using IBM SPSS Statistics (Version 27).

## 3 Results

Means and standard deviations of sensory processing, social camouflaging and ED symptoms are reported in Table 1.

Ward's HCA showed three distinct clusters (see Supplementary Materials 1). Of a total of 169 participants who had complete EDE‐QS data, 39 (23.1%) were in Cluster 1, 89 (52.7%) were in Cluster 2 and 41 (24.2%) were in Cluster 3. Cluster 1 was characterised by high social camouflaging behaviours but lower sensory processing differences (labelled *high camouflaging* (*z* = 4.3), *low sensory* (*z =* −1.18)). Cluster 2 was characterised by high social camouflaging behaviours and high sensory processing differences (labelled *high camouflaging*, (*z* = 0.49), *high sensory* (*z* = 0.58)). Cluster 3 was characterised by low social camouflaging behaviours and average sensory processing differences (labelled *low camouflaging* (*z =* −1.19), *average sensory* (*z* = −0.03)).

Clusters were tested for differences in age and gender, with no between‐cluster differences reported for age (*F*(2, 168) = 0.710, *p* = 0.493) or gender *χ* ^2^(6) = 12.39, *p* = 0.054). Clusters were then compared on EDEQ‐S scores. A one‐way ANOVA was conducted to explore differences between clusters on eating disorder symptoms, reporting a significant effect (*F*(2, 168), = 3.96, *p* = 0.021, *η*_p_^2^ = 0.046. Tukey's post‐hoc analyses reported a significant difference between Cluster 2 (*high camouflaging, high sensory:* mean = 15.11, standard deviation 9.4) and Cluster 3 (*low camouflaging, average sensory:* mean = 10.42, standard deviation = 9.1), with Cluster 2 reported significantly higher eating disorder symptoms than Cluster 3 (mean difference = 4.69, CI [0.62, 8.76], *p* = 0.02). Cluster 1 (*high camouflaging, low sensory*) also had slightly elevated ED symptoms (*M* = 12.3), although this difference was not statistically significant compared Cluster 2 (mean difference = −2.77, CI [−7.0, 1.44]) or Cluster 3 (mean difference = 1.91, CI [−3.0, 6.73]).

## 4 Discussion

This brief report found preliminary evidence for three distinct clusters in sensory processing and social camouflaging behaviours in a sample of Autistic adults. Over half of participants (∼53%) were in a group that exhibited both high social camouflaging behaviours and high sensory processing differences, and this group reported significantly higher ED symptoms than those in a cluster characterised by low social camouflaging behaviours and average sensory processing differences. Findings suggest that the combination of high social camouflaging and high sensory processing differences may place Autistic individuals at higher ED risk, although further research with larger, longitudinal datasets is urgently required before firm conclusions can be drawn.

Findings support previous literature that posits sensory processing (Brede et al. 2020; Cobbaert et al. 2024) and social camouflaging (Bradley et al. 2024; Nimbley, Buchan, et al. 2025) as important mechanisms underpinning the overlap between autism and EDs. This finding tentatively extends the current knowledge base, suggesting that it may be a *combination* of both these factors that leads to high ED risk in Autistic individual. This combination particularly concerning given that over half of the participants (∼53%, *n* = 89) fell into the ‘social‐sensory’ cluster and were thus in the higher risk group for ED symptoms. Just how these factors interact is an important avenue for future research. In line with previous qualitative research (Dean et al. 2017; Cook et al. 2022), it could be that experiencing sensory processing differences, or sensory overwhelm, may lead some Autistic individuals to engage in camouflaging behaviours. Future mixed‐method research, integrating sensory and social camouflaging outcomes with lived experience, with ED samples is thus warranted to explore how this is experienced and whether this process in turn increases the risk or severity of EDs. Such research would advance and extend our knowledge as to how this particular interaction of putative factors may constitute a pathway of risk or maintenance for the development of an ED in Autistic people.

Not only should future studies investigate *how* the mechanisms interact, but keen interest also lies in *when* these mechanisms interact, and when this becomes relevant to ED risk. Larger, longitudinal datasets would allow for the exploration of developmental trajectories and identification of possible ‘at risk’ ages or stages. This may be particularly relevant when considering social camouflaging and its importance as implicated in current findings. Peak age of onset of an ED is in adolescence (M. Solmi et al. 2022), a time when there are significant increases in social demands and greater focus on the importance of peer relationships and ‘fitting in’ (Dahl et al. 2018; Bagwell and Bukowski 2018), and may thus be a particularly important period of development for the influence of social camouflaging behaviours. A significant body of literature has explored eating behaviours in Autistic children, such as selective eating or fear of trying new foods (e.g., Baraskewich et al. 2021; Margari et al. 2020), with an increasing body of evidence supporting that these eating behaviours persist across development into adulthood (e.g., Schroder et al. 2022). For some Autistic individuals, these eating behaviours may develop into an eating disorder (Huke et al. 2013; Schroder et al. 2022), however it currently unclear why this may the case for some and not for others, as well as when Autistic individuals may be at particular risk of disordered eating behaviours developing. Future research should thus seek to explore the interaction between implicated factors, such as sensory processing and social camouflaging across development, seeking to identify at what point these two factors begin to interact and at what stage they may begin to increase risk for ED symptoms.

While the current study tentatively identified an ‘at risk’ cluster for ED symptoms in Autistic individuals, only two of a wide range of implicated factors were included in analyses. Future research should also consider the possible role of other potential mediators that were not included in the current analyses, such as depression (Adams et al. 2024) or alexithymia (Moseley et al. 2023), as well as the impact and timing of an autism diagnosis. ED clinicians report that the majority of Autistic adolescents they see are not diagnosed prior to attendance for ED treatment (Duffy et al. 2025), while receiving an early autism diagnosis has been reported to be a protective factor against the development of an ED (Brown et al. 2024). Thus, subsequent research should consider a wide range of factors, seeking to explore patterns or clusters across implicated mechanisms that might place the Autistic individual are greater risk of an ED. This would allow for the generation of more robust at‐risk profiles, and the potential to improve early detection and prevention of EDs for Autistic people. Thus, such understandings could provide an evidence base that can be used in the future to inform prevention or treatment strategies and improve outcomes quality of life for Autistic people with an ED, although the authors note that definitive clinical implications are beyond the scope of the current brief report.

Further limitations of the study include concerns surrounding the representativeness and generalisability of the sample. Participants were adults only and female‐biased, as well as biased towards white participants, echoing a lack of diversity observed across the broader autism (West et al. 2016) and eating disorder (Halbeisen et al. 2022) literature. Furthermore, an online study likely leads to the exclusion of those with low digital access, such as those from lower socio‐economic backgrounds, older adults or those with intellectual disabilities. Further research should draw on more accessible and inclusive research methods with more diverse, representative samples. Given the nature of the brief report, our approach was limited to examining whether clusters differed on eating disorder symptoms, which provides evidence of the clinical relevance of the identified groups. I did not, however, test for predictive validity of sensory processing and social camouflaging in determining cluster membership (e.g., Meneguzzo et al. 2025). As such, the present findings should be interpreted as demonstrating differences between clusters rather than indicating that cluster membership can be prospectively predicted from sensory processing or camouflaging variables. Future research should therefore examine predictive validity in larger and more diverse samples.

Despite these limitations, the current brief report found evidence for three distinct clusters of sensory processing and social camouflaging behaviours in a sample of Autistic adults, with tentative evidence to suggest a group high in both social camouflaging and sensory processing difficulties reporting higher ED symptoms. Findings suggest the combination of these factors may place Autistic individuals at higher ED risk, although future longitudinal, mixed‐method and more representative research, which considers a wider range of risk mechanisms, is urgently needed before conclusions can be drawn.

## Funding

E.N., M.S., H.S., F.D. and K.G.S. are funded by the UK Research and Innovation (UKRI), Medical Research Foundation (MRF) and National Institute for Health and Care Research (NIHR) through the Eating Disorder and Autism Collaborative (EDAC; Grant MR/X03058X/1). H.S. is also supported by the Medical Research Council/Arts and Humanities Research Council/Economic and Social Research Council Adolescence, Mental Health and the Developing Mind initiative as part of the EDIFY programme (Grant MR/W002418/1).

## Ethics Statement

Ethical approval was obtained from the School of Health in Social Science Research Ethics Committee at the University of Edinburgh (23‐24LCPS179).

## Consent

All participants provided informed consent to take part in the study.

## Conflicts of Interest

The authors declare no conflicts of interest.

## Supporting information


**Figure S1:** Dendrogram exploring clusters of social camouflaging behaviours and sensory processing differences.

## Data Availability

The data that support the findings of this study are available on request from the corresponding author and subject to future ethical approval. The data are not publicly available due to privacy or ethical restrictions.
