# Supplementary material for: Towards Identifying Autistic Adults at Risk for Eating Disorders: A Brief Report Into Clustering of Social Camouflaging and Sensory Processing Differences
Source: Eur Eat Disord Rev. 2025 Dec 2;34(3):648–54. doi: 10.1002/erv.70062 (PMC13048732; doi:10.1002/erv.70062)

**Supplementary Materials**

Supplementary Materials 1. *Dendrogram exploring clusters of social camouflaging behaviours and sensory processing differences.*


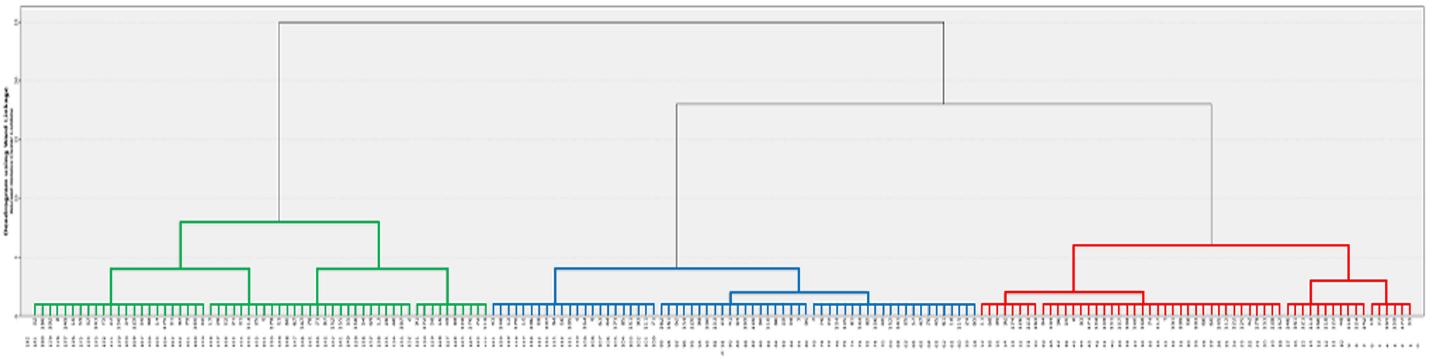

Supplement: Supplementary file 1 — Figure S1: Dendrogram exploring clusters of social camouflaging behaviours and sensory processing differences. [file ERV-34-648-s001.docx]
